# Hypertension in rats is associated with an increased permeability of the colon to TMA, a gut bacteria metabolite

**DOI:** 10.1371/journal.pone.0189310

**Published:** 2017-12-13

**Authors:** Kinga Jaworska, Tomasz Huc, Emilia Samborowska, Leszek Dobrowolski, Klaudia Bielinska, Maciej Gawlak, Marcin Ufnal

**Affiliations:** 1 Department of Experimental Physiology and Pathophysiology, Laboratory of Centre for Preclinical Research, Medical University of Warsaw, Warsaw, Poland; 2 Mass Spectrometry Laboratory, Institute of Biochemistry and Biophysics, Polish Academy of Sciences, Warsaw, Poland; 3 Department of Renal and Body Fluid Physiology, M. Mossakowski Medical Research Centre, Polish Academy of Sciences, Warsaw, Poland; 4 Laboratory of Physiology and Pathophysiology, Centre for Preclinical Research, Medical University of Warsaw, Warsaw, Poland; "INSERM", FRANCE

## Abstract

An increased blood trimethylamine N-oxide (TMAO) has emerged as a marker of cardiovascular mortality, however, the mechanisms of the increase are not clear. We evaluated if hypertension was associated with changes in the colon permeability to trimethylamine (TMA), a TMAO precursor. We did experiments on male, 24-26-week-old normotensive Wistar-Kyoto rats (WKY), spontaneously hypertensive rats (SHR) and SHR treated with enalapril, an antihypertensive drug (SHR-E). To check the colon permeability and liver TMA clearance, blood was collected from the portal vein and hepatic veins confluence, at baseline and after the intracolonic administration of TMA. Arterial blood pressure (BP) and intestinal blood flow (IBF) recordings and histological assessment of the colon were performed. SHR showed an increased gut-blood barrier permeability to TMA. Namely, at baseline SHR had a higher BP and portal blood TMA, but a lower IBF than WKY. After the intracolonic administration of TMA, SHR had a significantly higher portal blood TMA and higher TMA liver clearance than WKY. In SHR the arteriolar walls of the colon mucosa were significantly thicker than in WKY. Furthermore, SHR showed a significant decrease in the height of the mucosa. In contrast, SHR-E had lower portal blood TMA, lower BP and smaller thickness of arteriolar walls, but higher IBF than SHR, which indicates improved function of the gut-blood barrier in SHR-E. All groups had similar immunostaining of occludin and zonula occludens-1, markers of tight junctions. In conclusion, hypertensive rats show an increased permeability of the colon to TMA, which is accompanied by morphological and hemodynamic alterations in the colon. Therefore, cardiovascular diseases may be characterized by an increased permeability of the gut-blood barrier to bacterial metabolites such as TMA.

## Introduction

Increasing evidence suggests that gut microbiota produce biologically active compounds that enter the circulation and affect the circulatory system homeostasis [1–3]. To enter the circulation, gut bacteria metabolites need to pass the gut-blood barrier (GBB). The integrity and permeability of the GBB is dependent on numerous factors, including intestinal blood flow [4]. Hypertension is a major risk factor for heart failure, coronary artery disease and stroke, causing high morbidity and mortality. Hypertension is known to produce pathological changes in vasculature, such as microangiopathy in the retina, kidneys and in other organs [5]. However, there are scant data on the effect on hypertension on intestinal vasculature.

A positive correlation between an elevated fasting plasma trimethylamine N-oxide (TMAO), a gut bacteria metabolite, and an increased risk of major adverse cardiovascular events has been suggested [3, 6], however, the diagnostic value of blood TMAO level in cardiovascular diseases is debatable [7, 8].

In mammals, blood TMAO concentration increases after ingesting choline and L-carnitine which are absorbed from the small intestine. However, if the concentration of the nutrients exceeds the transport capacity of small intestine, they reach the large bowel and are metabolised by intestinal bacteria producing trimethylamine (TMA) [9, 10]. The bacteria-derived TMA is absorbed from the large bowel and goes with portal blood to the liver where it is oxidized to TMAO by flavin-containing monooxygenase-3 (FMO3) [11]. TMA and TMAO in humans are excreted mainly with the urine, but also with the sweat and the exhaled air [12, 13]. Therefore, the blood TMAO concentration may depend on several factors, including diet, gut microbiota activity, the GBB permeability to TMA, the oxidation of TMA by the liver, and TMA and TMAO excretion.

To the best of our knowledge, the effect of hypertension on the permeability of the colon, a major site of gut bacteria activity, has not been investigated. Therefore, in this study, we evaluated the permeability of the colon to TMA, as well as other factors that may influence blood TMA/TMAO concentration, including TMA liver clearance, TMA/TMAO excretion, and TMA stool concentration in normotensive and hypertensive rats.

## Materials and methods

The experiments were performed according to Directive 2010/63 EU on the protection of animals used for scientific purposes and approved by the I Local Bioethical Committee in Warsaw.

We did the study on male, 24-26-week-old, normotensive Wistar Kyoto (WKY, n = 30) rats, spontaneously hypertensive rats (SHR, n = 30) maintained on a standard laboratory diet and tap water, and on SHR, maintained on standard laboratory diet and treated with enalapril (Polpharma, Poland), an antihypertensive drug, dissolved in drinking water 100 mg/L (a dose of 12.9 ± 0.5 mg/kg BW) for 8 weeks (SHR-E, n = 30). Animals were provided by the Central Laboratory of Experimental Animals, Centre for Preclinical Research and Technology, Warsaw, Poland. All surgical procedures were performed under general anaesthesia with urethane (Sigma-Aldrich, Poland) at a dose of 1.5 g/kg of body weight (BW).

### Gut-blood barrier permeability

The experiments were performed on WKY (n = 12), SHR (n = 12) and SHR-E (n = 12). Rats were implanted with a polyurethane catheter inserted into the portal vein that collects blood from the intestines. Blood samples from the portal vein were collected at baseline and 30 and 60 min after the intracolonic administration of TMA (TMA intracolonic challenge test) or 0.25 mL of 0.9% NaCl saline (controls). Namely, half of the rats from the WKY, SHR and SHR-E groups were given saline and the other half were given TMA (100 mg/kg BW) dissolved in 0.25 mL of saline via a flexible polyurethane tube inserted 9 cm from the external anal sphincter.

### Liver clearance of TMA

The experiment was performed on WKY (n = 6), SHR (n = 6) and SHR-E (n = 6) rats implanted with polyurethane catheters inserted into the portal vein and into the inferior vena cava, just above the hepatic veins confluence. Blood samples from the veins were collected 30 min after the intracolonic administration of TMA (100 mg/kg BW) as described above.

TMA liver clearance was defined as the difference between portal blood TMA and hepatic vein blood TMA, and as the ratio of hepatic vein blood TMA and portal blood TMA, (1-$\frac{hepatic\;vein\;TMA}{portal\;vein\;TMA}$).

### Assessment of blood pressure and intestinal blood flow

WKY (n = 6), SHR (n = 6), and SHR-E (n = 6), were implanted with a polyurethane arterial catheter inserted into the aorta via femoral artery to measure blood pressure (BP) with Biopac MP 150 unit (Biopac Systems, Goleta, USA). Next, the upper mesenteric vein collecting the blood from the colon was separated from the surrounding tissue for a distance of ca. 10 mm, to enable placement of a flow probe (ID 1 mm). The access to the vein was obtained by performing midline laparotomy with a cut from the xiphoid to the navel. The intestines were lined outside the abdominal cavity and wrapped with moistened gauze, protecting them from drying. Measurements of upper mesenteric vein blood flow (intestinal blood flow—IBF) were conducted using the transit time ultrasound set-up which consists of a noncannulating acoustic (20 kHz) flow probe connected with a dedicated flowmeter (type T106, Transonic System Inc., Ithaca, N.Y., USA). The IBF and BP were measured at baseline, for 30 minutes after the intracolonic administration of 0.25 mL of saline, and 30 min after the intracolonic injection of TMA (100 mg/kg BW) dissolved in 0.25 mL of saline.

### 24-hr TMA balance, metabolic and biochemical data

WKY (n = 6), SHR (n = 6), and SHR-E (n = 6) were maintained for 2 days in metabolism cages to evaluate 24-hr TMA, water, food balance and to collect urine for TMA excretion study. Samples from the second day were analysed. After the experiments, blood from the right ventricle of the heart, stool, and intestine samples were collected as described below.

#### Blood biochemical tests

Rats were anaesthetized with urethane at a dose of 1.5 g/kg BW. Next, blood was taken to the chilled EDTA tubes. The sample was centrifuged for 5 minutes at 5000 rpm at 4°C. The plasma was collected into Eppendorf tubes and frozen at -20°C. Biochemical blood analyses (creatinine, urea, AST, ALT) were performed on the *Cobas 6000 analyzer* (Roche Diagnostics, Indianapolis, USA).

#### Evaluation of TMA, TMAO and indoxyl sulfate concentration

Blood plasma, urine and stool concentration of TMA/TMAO and indoxyl sulfate was evaluated using liquid chromatography coupled with triple-quadrupole mass spectrometry as previously described [1]. Instrumentation consisted of Waters Acquity Ultra Performance Liquid Chromatograph coupled with Waters TQ-S triple-quadrupole mass spectrometer. The mass spectrometer operated in the multiple-reaction monitoring (MRM)- positive electrospray ionization (ESI) mode. The ion transitions were m/z 76.076> 57.97 and m/z 76.076> 58.97 for TMAO, m/z 60.08> 44.05 and m/z 60.08> 45.057 for TMA, m/z 64.09> 48.05 for TMA-13C315N, m/z 85.13> 68.2 for TMAO-D9, 212.00>79.96 and 212.00>132.04 for indoxyl sulfate and 216.11>135.76 for indoxyl sulfate-D4. Only first was used as quantification transition. The calibration curve ranges were 0.1–60 μg/mL for TMAO, 0.99–120 μg/mL for TMA and 0.1–50 μg/mL for indoxyl sulfate. The limits of quantification (LOQ) were 0.1 μg/mL, 0.99 μg/mL and 0.1 μg/mL for TMAO, TMA and indoxyl sulfate, respectively.

#### Preparation of stool samples for TMA and TMAO analysis

After the blood taking, anaesthetized rats were killed by decapitation. A 5–6 cm long segment of the colon (a middle part between the cecum and the rectum) was closed with sutures and removed. The isolated fragment of the colon was dissected and the tissues were used for histological and immunohistochemical evaluation.

A sample of 0.5 mL of stools from the removed colon was weighted and homogenized with 1.0 mL of 0.9% NaCl in a closed 2 mL laboratory tube by vortexing it for 5 min. Afterwards, the sample was centrifuged for 5 minutes at 5000 rpm, and 1 mL of the obtained supernatant was transferred to a tube with a 0.45 μm pore size filter (Ultrafree-CL, Merck KgaA, German) and again centrifuged for 5 minutes at 5000 rpm. All procedures were performed at the temperature of 2–5°C. The supernatant was collected into Eppendorf tubes and frozen at -20°C.

### Histology and morphometry of the colon

Histological assessment was performed on tissues harvested from WKY, SHR and SHR-E. Tissues sections fixed in 10% buffered formalin were dehydrated by means of graded ethanol and xylene baths and embedded in paraffin wax. Sections of 3–4 μm were stained with haematoxylin and eosin (HE) and van Gieson stain (for connective tissue fibres). General histopathological examination was evaluated at magnification of 10x, 40x and 100x (objective lens) and 10x (eyepiece) and photographic documentation was made. The mucosa and submucosa of the colon, intestinal crypts and its cell composition, blood vessels of mucosa and submucosa of the colon were examined. The morphometric analysis included: the height of the mucous membrane—measured at magnification of 10x (objective lens) and 10x (eyepiece), and the thickness of walls of arterioles which were visible in submucosa—measured at magnification of 40x (objective lens) and 10x (eyepiece). Measurements of the width of the vascular wall were made for two types of vessels: in diameter 25–50 μm—smaller arterioles or in diameter 75–100 μm larger arterioles. Both types of arterioles were in the colonic submucosa. The height of the colonic mucosa was measured from the top of the crypt to the beginning of the muscularis mucosae. The microscopic evaluation was performed in a blinded fashion, using a standard light microscope Olympus BX41 and CellSens software (Olympus Corporation, Tokyo, Japan).

### Immunohistochemistry staining of occludin and ZO-1

Tissue was fixed in 4% paraformaldehyde in PBS (phosphate-buffered saline) (4°C, 24 hours), impregnated with 30% sucrose solutions (w/v in PBS) and frozen in dry-ice cold heptane. Next, the tissue was cut with cryotome into 12 μm thick sections and mounted on adhesion slides (Superfrost™ Ultra Plus, Thermo Scientific).

Sections were blocked with 5% goat serum in PBST (phosphate-buffered saline with 0.1% Triton X-100) and incubated overnight at 4°C with primary antibodies (anti-ZO-1, Thermo Fisher, catalog number: 61–7300 or anti-occludin, Thermo Fisher, catalog number: 71–1500, Waltham, USA). Next, sections were washed with PBST and incubated with Cy3-conjugated goat anti-rabbit IgG (Jackson ImmunoResearch Laboratories, catalog number: 111-165-144) secondary antibody at room temperature for 2 h. After washing with PBST, mounting medium (Vectashield with DAPI, Vector, catalog number: H-1200) sections were coverslipped. Anti-ZO-1 staining or anti-occludin staining of all three experimental groups were processed simultaneously.

Immunofluorescence measurements were performed with an Olympus X1000 confocal microscope (Olympus Corporation, Tokyo, Japan), 20x objective. The same parameter settings for all three experimental groups were applied for measurements of anti-ZO-1 staining signal or anti-occludin staining signal. Images were analyzed with Fiji software [14]. Mean signal of fluorescence was measured for ROIs (regions of interest) which were manually selected within epithelial cells of the mucous membrane of the colon. Background signal was measured in area of slide with no tissue and was subtracted from epithelium fluorescence signal.

### Statistical analyses

The Kolmogorov-Smirnov test was used to test normality of the distribution. Arterial blood pressure (BP) and heart rate (HR) were calculated by AcqKnowledge 4.3.1 Biopac software (Biopac Systems, Goleta, USA). For evaluation of systolic and diastolic BP, HR and IBF within the series, the average over 5-minute before intracolonic infusion was compared with the averages over 5 minutes after the intracolonic infusion by ANOVA for repeated measures. Differences between the groups were evaluated by one-way or multivariate ANOVA, followed by Tukey’s post hoc test. A value of two-sided p<0.05 was considered significant. Analyses were conducted using STATISTICA 12.0 (Stat Soft, Krakow, Poland).

## Results

### Gut-blood barrier permeability

#### Gut-blood barrier permeability at baseline

At baseline, spontaneously hypertensive rats (SHR, n = 12, 4.20±0.19 μg/mL) had a significantly higher portal blood TMA than normotensive Wistar Kyoto rats (WKY, n = 12, 3.09 ± 0.25 μg/mL) and SHR treated with enalapril, an antihypertensive drug (SHR-E, n = 12, 3.11 ± 0.21 μg/mL), (p<0.05). In addition, SHR (5.95 ± 0.62 μg/mL) had a significantly higher hepatic blood indoxyl sulfate level than WKY (3.55 ± 0.29 μg/mL) and SHR-E (4.49 ± 0.44 μg/mL), (p<0.05).

#### TMA intracolonic challenge test

**Fig 1:**
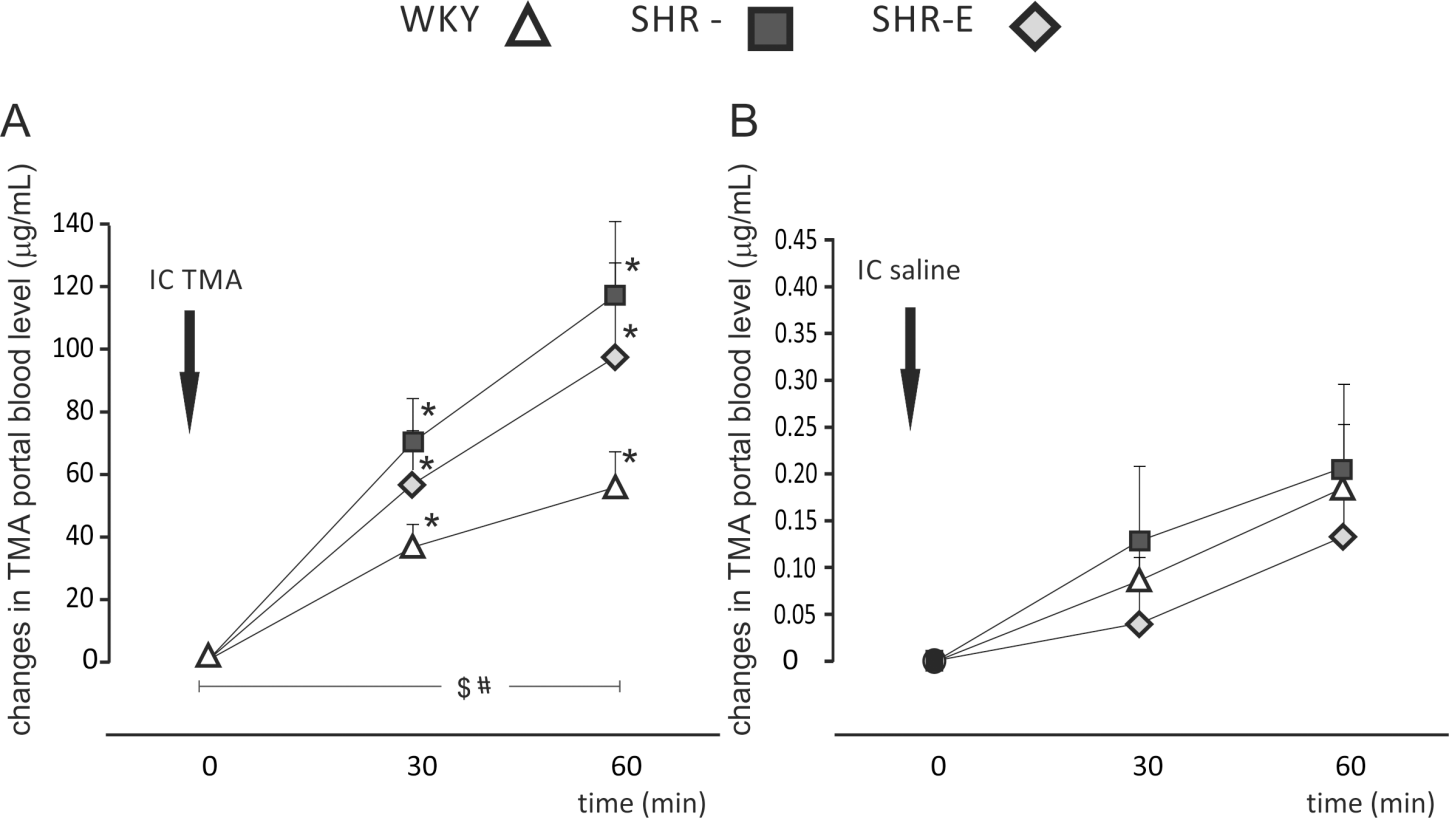
TMA intracolonic challenge test. Changes in trimethylamine (TMA) portal blood level (μg/mL) after the intracolonic administration of **A)** TMA (100 mg/kg BW) dissolved in 0.25 mL of 0.9% NaCl (IC TMA) or **B)** 0.25 mL of 0.9% NaCl (IC saline) in normotensive Wistar Kyoto rats (WKY) (n = 6), spontaneously hypertensive rats (SHR) (n = 6), and enalapril-treated SHR (SHR-E) (n = 6). Values are means, + SE, *—p<0.05 vs baseline, ^$^- p<0.05 WKY vs SHR, ^**#**^- p<0.05 WKY vs SHR-E (repeated measures ANOVA followed by post hoc Tuckey test).

At baseline, SHR (n = 6) had a significantly higher portal blood TMA than WKY (n = 6) and SHR-E (n = 6), i.e. SHR: 4.23 ± 0.35 μg/mL, WKY: 2.69 ± 0.20 μg/mL, and SHR-E: 3.05 ± 0.20 μg/mL, (p<0.05). The intracolonic administration of TMA produced a significant increase in portal blood TMA in all the groups [SHR (p<0.05), WKY (p<0.05), SHR-E (p<0.05), by one-way ANOVA for repeated measures]. The increase in the SHR group was significantly higher than in WKY and SHR-E groups (p<0.05, by ANOVA for repeated measures, followed by post-hoc Tuckey test), (Fig 1A). The intracolonic administration of saline did not produce a significant change in portal blood TMA (Fig 1B).

### Liver clearance of TMA

After the intracolonic administration of TMA, SHR had a significantly higher portal blood TMA than WKY and SHR-E (Table 1). TMA liver clearance was defined as the difference between portal blood TMA and hepatic vein blood TMA, and as the ratio of hepatic vein blood TMA and portal blood TMA (1-$\frac{hepatic\;vein\;TMA}{portal\;vein\;TMA}$). SHR had a significantly higher TMA liver clearance in comparison to WKY and SHR-E, (Table 1).

**Table 1:** TMA and TMAO liver clearance.

Group/ parameter	WKY (n = 6)	SHR (n = 6)	SHR-E (n = 6)	ANOVA
Portal vein TMA (μg/mL)	41.84 ± 5.78	71.34 ± 5.67	42.1 ± 5.52	(F_2,15_ = 10.1, p<0.05),*^,^^†^
Hepatic vein TMA (μg/mL)	27.68 ± 3.31	29.17 ± 2.52	25.6 ± 3.66	NS
Liver TMA clearance (Portal—Hepatic veins difference) (μg/mL)	14.15 ± 4.06	42.17 ± 5.98	16.5 ± 6.31	(F_2,15_ = 8.2, p<0.05),*^,^^†^
Liver TMA clearance (1-$\frac{hepatic\;vein\;TMA}{portal\;vein\;TMA}$).	0.32± 0.05	0.58 ± 0.04	0.33 ± 0.10	(F_2,15_ = 3.9, p<0.05),*^,^^†^
Hepatic vein TMAO (μg/mL)	5.61 ± 0.41	15.27 ± 1.24	10.86 ± 1.73	(F_2,15_ = 12.8 p<0.05),*^,^^‡^

Portal and hepatic vein blood TMA and TMAO (μg/mL) 30 min after the intracolonic administration of TMA (100 mg/kg BW) dissolved in 0.25 mL of 0.9% NaCl in Wistar Kyoto rats (WKY), spontaneously hypertensive rats (SHR), and enalapril-treated SHR (SHR-E). Values are means, ± SE. One-way ANOVA followed by post-hoc Tuckey-test. NS—not significant differences between groups.

*- p<0.05 for WKY vs SHR

^†^- p<0.05 for SHR vs SHR-E

^‡^- p<0.05 for WKY vs SHR-E.

### Assessment of blood pressure and intestinal blood flow

**Fig 2:**
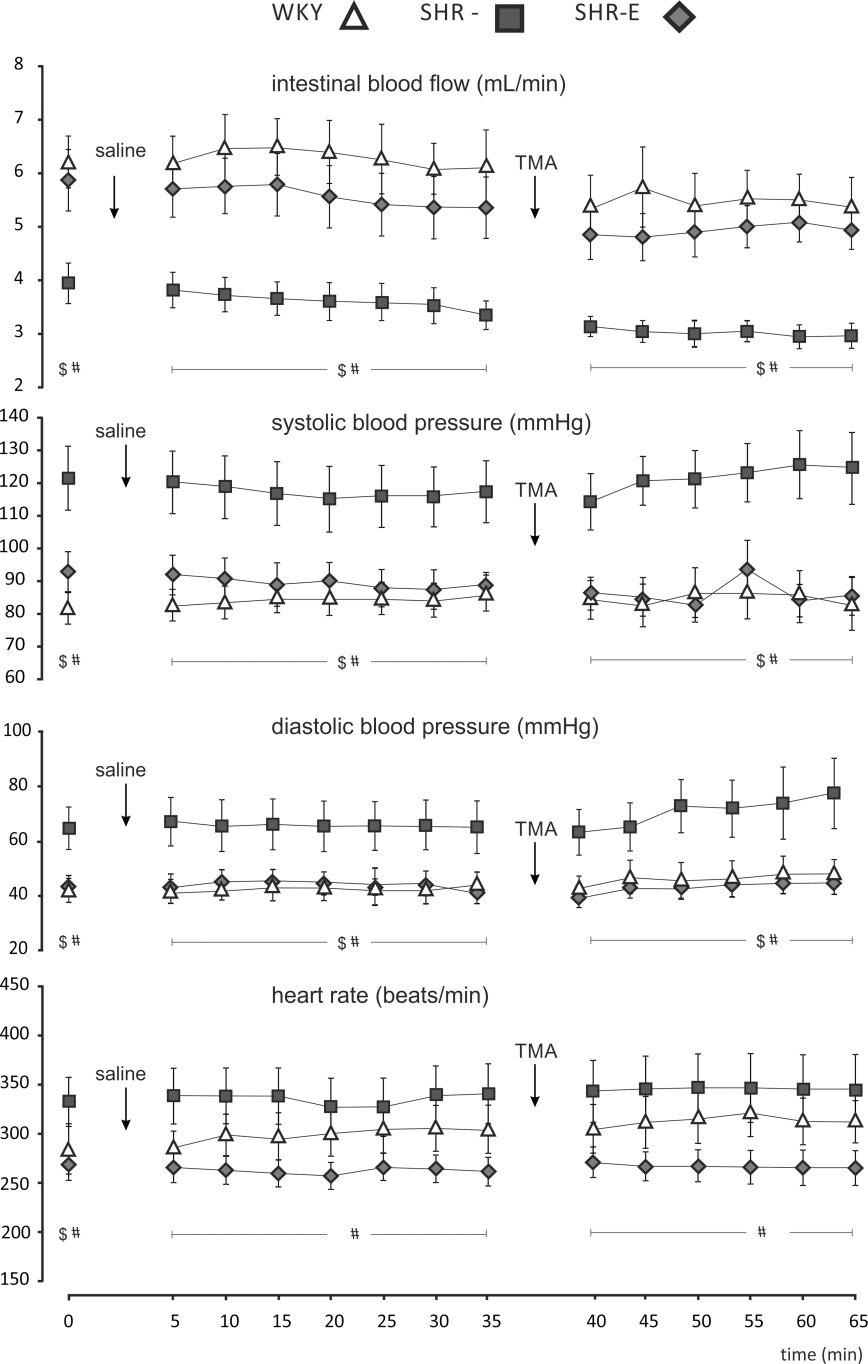
Assessment of intestinal blood flow and blood pressure. Intestinal (upper mesenteric vein) blood flow, systolic arterial blood pressure, diastolic arterial blood pressure and heart rate in anaesthetized normotensive Wistar-Kyoto rats (WKY) (n = 6), spontaneously hypertensive rats (SHR) (n = 6) and enalapril-treated SHR (SHR-E) (n = 6) at baseline (0) and after the intracolonic administration of 0.25 mL of 0.9% NaCl (↓ saline) and TMA (100 mg/kg BW) dissolved in 0.25 mL of 0.9% NaCl (↓ TMA). ^$^- p<0.05 for SHR vs WKY, ^#^- p<0.05 for SHR vs SHR-E comparison.

At baseline, there were significant differences between WKY, SHR and SHR-E in intestinal blood flow (IBF) (p<0.05), systolic blood pressure (SBP) (p<0.05), diastolic blood pressure (DBP) (p<0.05) and heart rate (HR) (p<0.05) as analysed with one-way ANOVA. Namely, SHR had a significantly lower IBF but higher SBP, DBP and HR in comparison to WKY and SHR-E (Fig 2). Such between-group differences were also present after the intracolonic administration of saline (p<0.05, for IBF), (p<0.05, for SBP), (p<0.05, for DBP) and (p<0.05 for HR) as analysed with ANOVA for repeated measures. Similar between-group differences were found after the intracolonic administration of TMA (p<0.05, for IBF), (p<0.05, for SBP), (p<0.05, for DBP) and (p<0.05, for HR), (Fig 2). There was no significant within-group difference in neither of the parameters after the intracolonic infusion of saline and TMA as analysed for 5 min pre-administration vs 20 min post-administration period by ANOVA for repeated measures. However, in all the groups there was a mild, non-significant decrease in IBF during the experiment.

### 24-hr TMA balance, metabolic and biochemical data

There were no significant differences between WKY, SHR and SHR-E in body mass and food intake. SHR-E showed a higher water intake and 24-hr urine output in comparison to WKY and SHR. There was a significant within group variation in stool TMA level (TMA concentration in stool sample extracts in 3 of 6 WKY, in 1 of 6 SHR and 2 of 6 SHR-E was below the limit of quantification), consequently statistical analysis showed no significant differences between the groups, (Table 2).

**Table 2:** Metabolic data and TMA/TMAO excretion.

Group/ Parameter	WKY (n = 6)	SHR (n = 6)	SHR-E (n = 6)	ANOVA
Stool TMA (μg/mL)	1.62 ± 0.16	2.04 ± 0.66	2.46 ± 0.81	NS^1^
Stool TMAO (μg /mL)	LQQ	LQQ	LQQ	-
Stool density (g/mL)	1.13 ± 0.09	1.06 ± 0.13	1.15 ± 0.08	NS
24-hr urine TMA output (μg)	137.0 ± 15.9	160.6 ± 21.4	161.6 ± 44.0	NS
24-hr urine TMAO output (μg)	436.8 ± 88.2	474.0 ± 74.4	426.6 ± 90.0	NS
Blood creatinine (mg/dL)	0.41 ± 0.05	0.45 ± 0.03	0.76 ± 0.04	(F_2,15_ = 15.2, p<0.05)^†^^,^^‡^
Blood urea (mg/dL)	38.8 ± 1.9	52.5 ± 1.6	63.0 ± 1.44	(F_2,15_ = 46.1, p<0.05)*^,^^†^^,^^‡^
AST (U/L)	83.0 ± 6.7	134.2 ± 21.7	214.6 ± 34.7	(F_2,15_ = 7.6, p<0.05)*
ALT (U/L)	51.7 ± 7.4	79.0 ± 14.9	99.6 ± 13.4	NS
Body mass (g)	344.45 ± 4.9	347.9 ± 13.4	359.6 ± 5.9	NS
24-hr water intake (mL)	25.6 ± 1.4	28.3 ± 1.4	46.2 ± 2.1	(F_2,15_ = 41.3, p<0.05)^†^^,^^‡^
24-hr food intake (g)	20.2 ± 0.9	20.3 ± 0.8	20.8 ± 0.5	NS
24-hr urine output (mL)	10.7 ± 0.7	8.6 ± 0.8	22.9 ± 1.7	(F_2,15_ = 44.2, p<0.05)^†^^,^^‡^

Metabolic data and TMA/TMAO excretion in normotensive Wistar Kyoto rats (WKY), spontaneously hypertensive rats (SHR), and enalapril-treated SHR (SHR-E). Values are means, ± SE. ANOVA followed by post-hoc Tuckey-test. NS–not significant differences between the groups.

^1^ –WKY (n = 3), SHR (n = 5), SHR-E (n = 4), TMA stool level in the remaining samples WKY (n = 3), SHR (n = 1), SHR-E (n = 2) was below the limit of quantification (LQQ).

*- p<0.05, WKY vs SHR

^†^- p<0.05, SHR vs SHR-E

^‡^- p<0.05, WKY vs SHR-E

There was no significant difference in peripheral blood TMAO level (blood taken from the right ventricle of the heart) between the groups (WKY: 0.17 ± 0.02 μg/mL, SHR: 0.23 ± 0.03 μg/mL and SHR-E: 0.19 ± 0.03 μg/mL), while blood TMA level was below the limit of quantification. 24-hr urine TMAO and TMA output was higher in SHR than in WKY, however, the difference was not significant. Plasma creatinine and urea were higher in SHR and SHR-E than in WKY. AST and ALT levels were higher in SHR-E rats in comparison to WKY, (Table 2).

### Histology and morphometry of the colon

**Fig 3:**
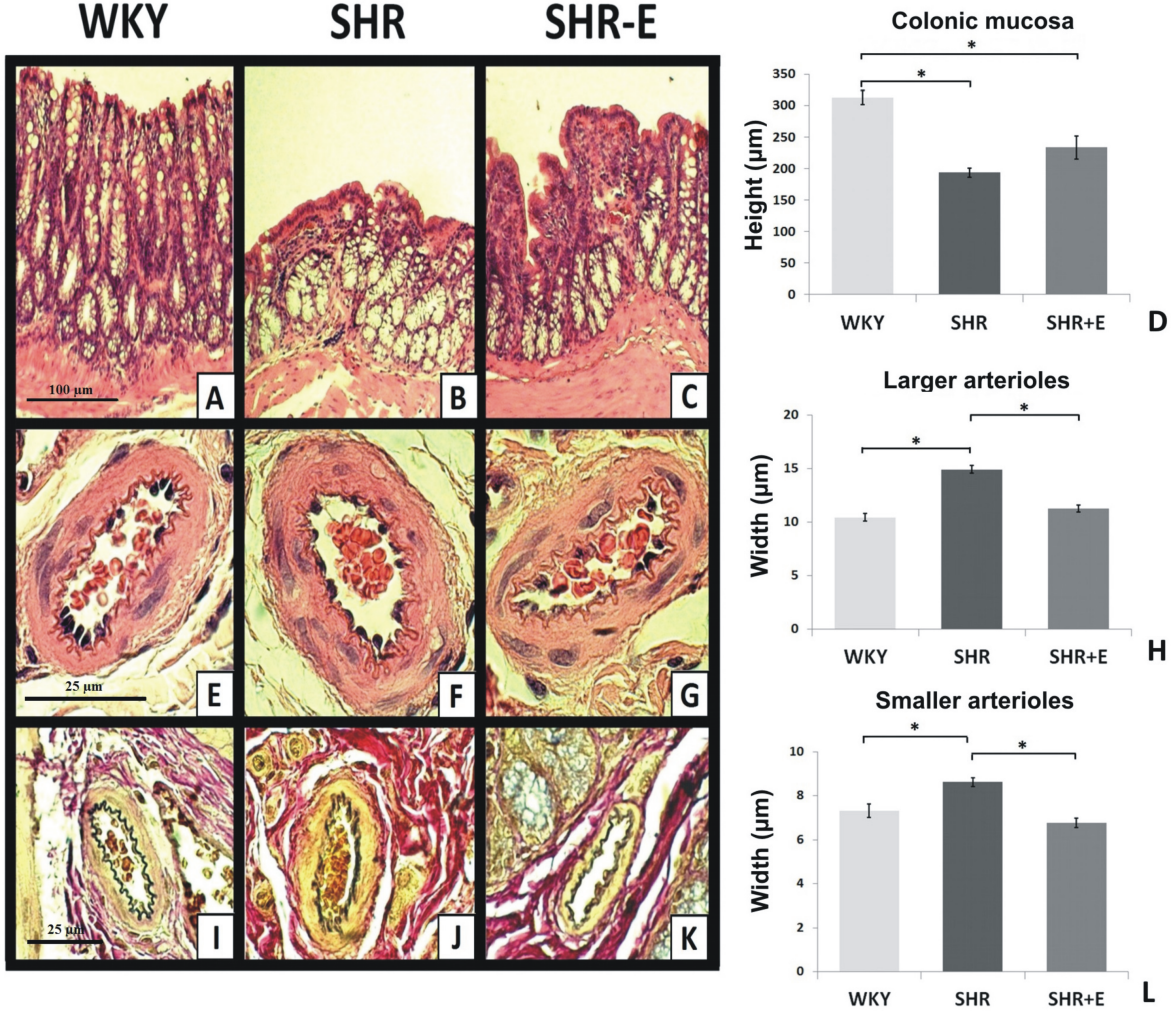
Histopathological and morphometric parameters in the colon of WKY (Wistar Kyoto rats), SHR (spontaneously hypertensive rats) and SHR-E (enalapril-treated SHR). **A, B, C–**The mucosa of the colon with haematoxylin and eosin (x10 objective lens). **E, F, G–**Larger arterioles of mucosa with haematoxylin and eosin (x100 objective lens). **I, J, K–**Larger arterioles of mucosa with van Gieson stain (x100 objective lens). **D–**The height of the colonic mucosa– μm (x10 objective lens). **H–**The width of the tunica media of larger arterioles– μm (x40 objective lens). **L—**The width of the tunica media of smaller arterioles– μm (x100 objective lens). *- p<0.05.

In SHR (n = 6), the walls of larger arterioles in the mucosa and submucosa membrane were significantly thicker than in WKY (n = 6) and SHR-E (n = 6), (p<0.05). Similar changes were found in smaller arterioles (p<0.05). In general, in the SHR group the lumen of arterioles was smaller, while the veins were more expanded and filled with blood in comparison to WKY and SHR-E, (Fig 3).

**Fig 4:**
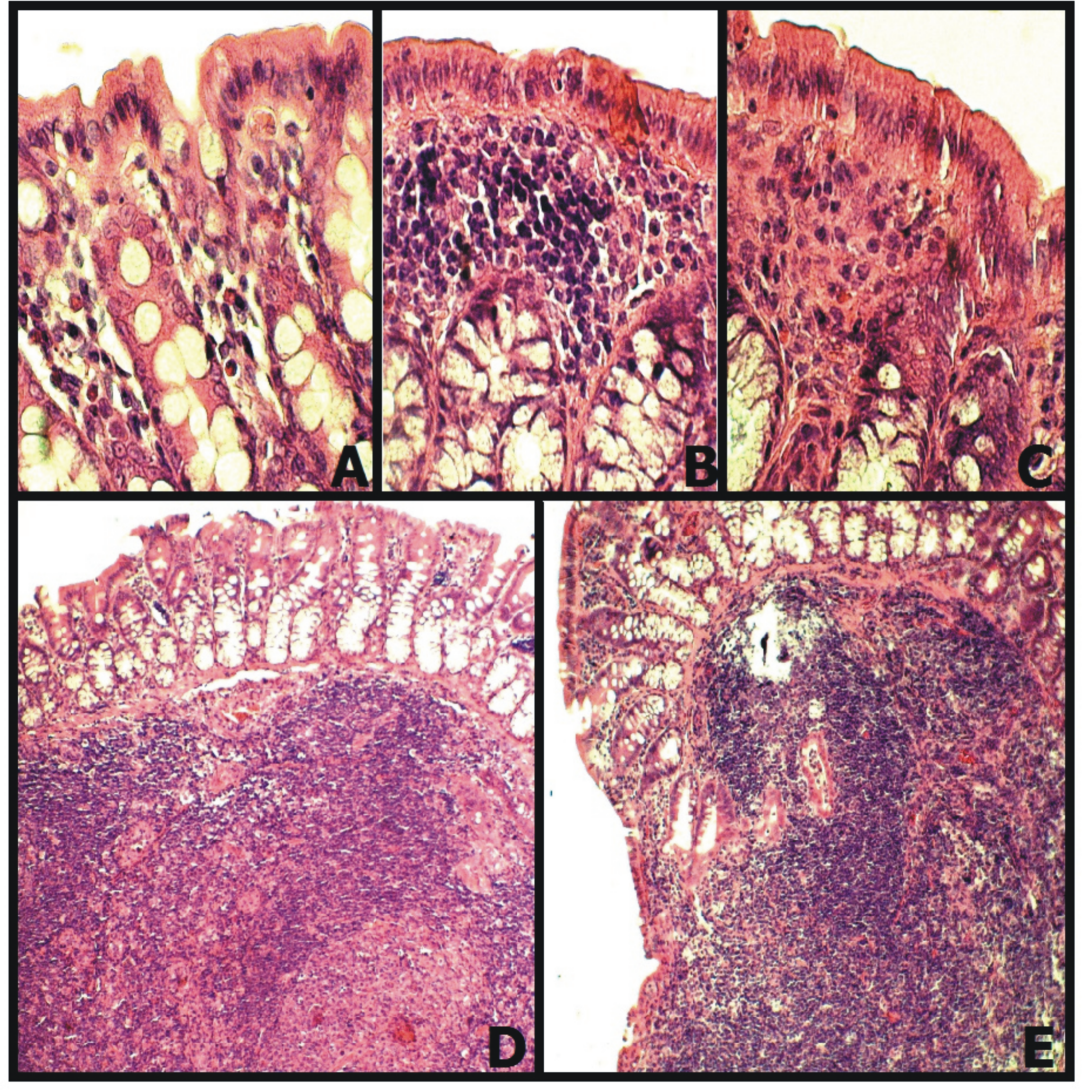
Inflammatory changes in the mucosa of the colon in WKY (normotensive Wistar Kyoto rats), SHR (spontaneously hypertensive rats) and SHR-E (SHR treated with enalapril). **A, B, C**–The colonic mucosa in WKY, SHR and SRE-E, respectively, (x 40 objective lens). **B**—Significant infiltration of mononuclear cells under the epithelium, similar to cryptopatches, (x 40 objective lens). **D, E**—Active GALT lymph nodes (Peyer's patches) were present only in SHR, (x 4 objective lens).

Morphometric measurement showed a significant decrease in the height of the mucosa in SHR (p<0.05). SHR showed also fewer goblet cells which were present only in the lower part of the intestinal crypts and had no contact with the light of the colon. There were no significant differences between SHR and SHR-E in the height of mucosa. SHR-E showed a small increase in the number of goblet cells in comparison to SHR, (Figs 3 and 4).

In SHR in the van Gieson staining, the hyperplasia of connective tissue of mucosa and submucosa was observed. There was no fibrosis in the walls of arterioles, however, the increase in thickness of adventitia and the amount of connective tissue around arterioles were present, (Fig 3). Besides, SHR showed infiltration of mucosa by mononuclear cells and their clusters under the epithelium. Additionally, numerous lymphoid follicles of GALT (*gut-associated lymphoid tissue*) and cryptopatches were found, but there was no increased infiltration of epithelium by migrating lymphocytes or other leukocytes (Fig 4).

### Immunohistochemistry studies of occludin and ZO-1

**Fig 5:**
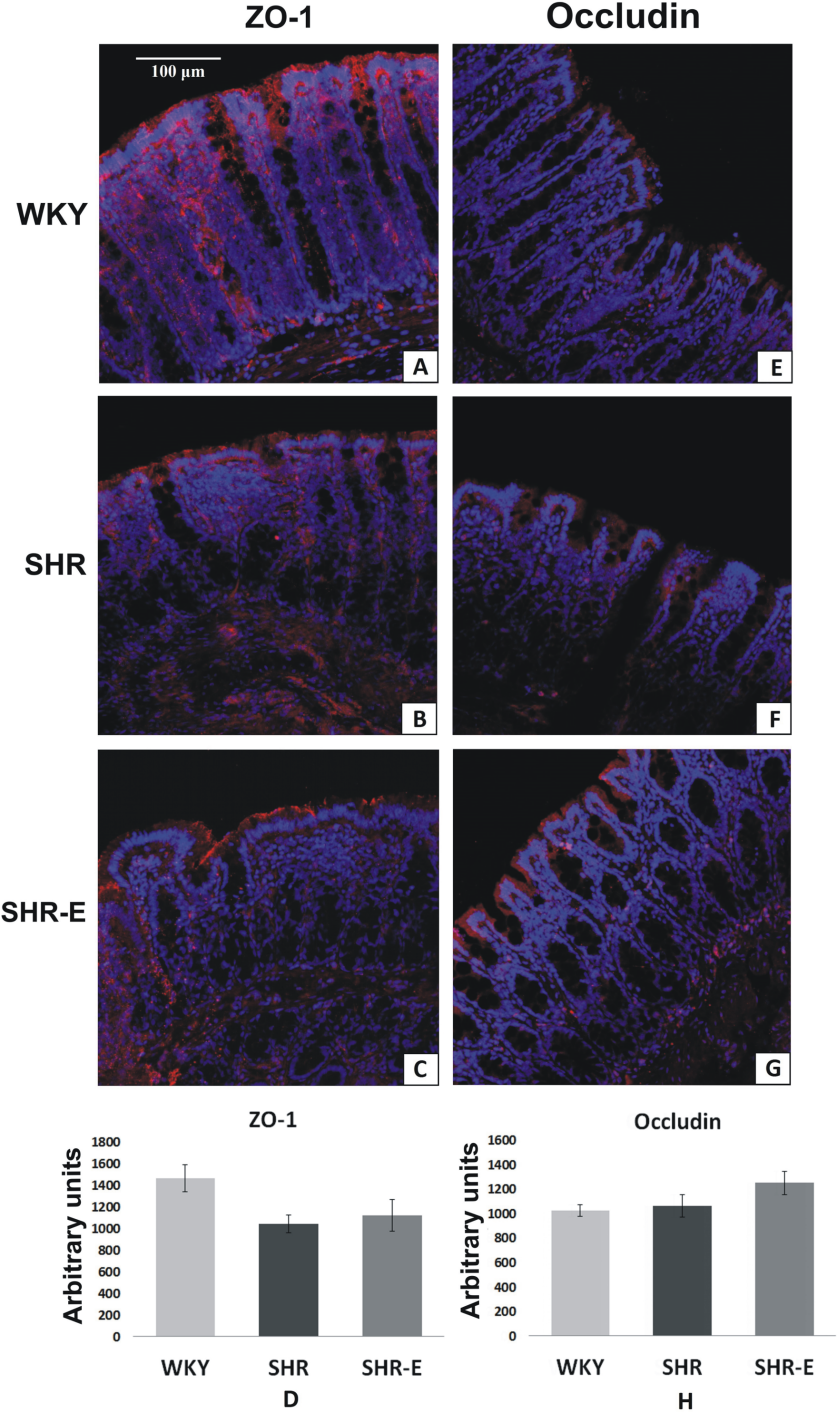
Immunohistochemistry stain of zonula occludens-1 (ZO-1) and occludin in colon mucosa. **A, B, C**–The colonic mucosa with the immunostaining of ZO-1 in WKY (Wistar Kyoto rats), SHR (spontaneously hypertensive rats) and SHR-E (enalapril-treated SHR). **E, F, G—**The colonic mucosa with the immunostaining of occludin in WKY, SHR and SHR-E. **D, H–**Changes of mean signal of fluorescence in epithelium of the colonic mucosa (arbitrary units of fluorescence).

There was no significance difference between the groups in mean fluorescent signal for ZO-1 and occludin. However, there was a tendency for higher values for ZO-1 staining in WKY than in SHR and SHR-E (p = 0.13, by one-way ANOVA), (Fig 5).

## Discussion

A new finding of our study is that hypertensive rats show an increased permeability of the colon to TMA, a gut bacterial metabolite and the precursor of TMAO. This suggests that hypertension facilitates penetration of gut microbiota metabolites to the circulation, and that diagnostic value of blood TMAO level, a new marker of cardiovascular risk, may depend on the colon permeability.

Gut bacterial metabolites such as H_2_S and TMAO affect the functions of the circulatory system [1, 15]. Recent clinical studies showed a positive correlation between an elevated blood TMAO and an increased cardiovascular risk [3, 10, 16] and some experimental evidence shows potentially negative effects of TMAO on the circulatory system [3, 17]. Since TMAO is a metabolite of phosphatidylcholine and L-carnitine, TMAO has been proposed to constitute a link between the diet and cardiovascular diseases [10]. However, due to high interindividual variety of blood TMAO [8, 10] and the beneficial role of TMAO in some animals [18], the relevance of TMAO as a diagnostic marker and/or a causative factor in cardiovascular diseases is debatable [7]. Furthermore, the mechanism of an increased blood TMAO in cardiovascular diseases has been not elucidated.

Blood TMAO level may depend on numerous factors such as gut microbiota activity (production of TMA, a TMAO precursor), the GBB permeability to TMA, the oxidation of TMA by the liver and the excretion of TMA and TMAO [19]. In general, there are scant data on TMA/TMAO balance in mammals, and, to the best of our knowledge, the influence of hypertension on the factors affecting TMAO blood level has not yet been studied.

The access of TMA and other gut bacteria products to the circulation is protected by the GBB, a complex multilayer system [20, 21]. Interestingly, there is some clinical evidence that cardiovascular diseases such as heart failure may impair the function of the GBB [22]. Recently, Santisteban at al. found that hypertensive rats showed morphological changes in the small intestine and higher systemic blood level of orally administered 4kDa dextran [23]. However, systemic blood concentration of orally given compounds depends on numerous factors including gastrointestinal motility, intestinal absorption, liver metabolism, and kidney excretion. Furthermore, dextran is an exogenous compound, therefore changes in the gut permeability to dextran is of unknown physiological and pathological significance. Therefore, in this study we measured portal blood concentration of TMA, a mammalian gut bacteria-derived compound. This approach has allowed us to asses directly the GBB permeability, excluding the abovementioned confounding factors. In addition, we measured hepatic vein blood concentration of indoxyl sulfate, a gut bacterial metabolite of tryptophan [24].

Our study shows that SHR had a significantly higher blood TMA and indoxyl sulfate levels than WKY at baseline. Furthermore, SHR had a significantly higher portal blood TMA than WKY after the intracolonic TMA challenge test. These findings suggest that hypertension increases the colon permeability to gut bacterial metabolites.

It has been found that functions of the GBB are compromised by decreased IBF [25–27]. In our study, in comparison to normotensive WKY, hypertensive SHR had a decreased IBF and decreased height of the mucosa. The latter was the likely cause of an increased permeability of the colon to TMA in SHR. Decreased IBF in hypertensive rats was also reported by others [28]. Furthermore, in our study SHR showed a significant increase in the thickness of arterioles walls, resulting from hyperplasia of smooth myocytes in the tunica media and from the hyperplasia of connective tissue of the adventitia. Such changes usually reflect the adaptation of arterioles to elevated blood pressure. Thicker walls and reduced lumen of arterioles increase the vascular resistance and could be responsible for a decreased IBF in SHR. Additionally, we found a decreased number of goblet cells, increased inflammatory cells infiltration and GALT stimulation in the SHR’s colonic mucosa. The similar histological findings were reported in the small intestine [23].

In comparison to SHR, SHR treated with enalapril (SHR-E) showed a significantly lower portal blood TMA level at baseline, and a trend for lower portal blood TMA level in the intracolonic TMA challenge test. Furthermore, SHR-E showed a lower arterial blood pressure, higher IBF and smaller morphological changes in the colon than SHR. This may suggest that enalapril treatment improved the GBB function by reducing the hypertension-induced hemodynamic and morphological changes in the colon.

The mean of gut bacterial metabolites transportation through the gut’s wall is unknown. The regulation of the paracellular transport is provided by tight junctions consisting of several transmembrane and cytoplasmic proteins, such as occludin and zonula occludens-1 (ZO-1) [29], which are often used as tight junctions markers [30]. In our study, SHR and WKY had a similar immunofluorescent signal of occludin and ZO-1 suggesting no significant effect of a decreased intestinal blood flow on tight junction composition with regard to occludin and ZO-1 level. Therefore, we think that an increased permeability of the colon to gut bacterial metabolites in SHR was caused by the reduced height of the colon mucosa, a major structural and functional layer of the GBB, rather than by alterations in tight junctions.

Another factor that may affect blood concentration of gut bacterial metabolites is gut microbiota metabolic activity. Here, we attempted to check the TMA concentration in SHR and WKY stools, but our results do not allow to draw clear conclusions. The processing of the stools reduced the concentration of TMA, a strongly volatile compound. Therefore, in several samples, mostly in WKY, the TMA level was below the limit of quantification. Further studies are needed to elucidate this issue.

Finally, we have found that SHR had a higher TMA liver clearance than WKY. Namely, after the intracolonic administration of TMA, more TMA was converted to TMAO by SHR than by WKY. This may suggest higher activity of enzymes oxidizing TMA to TMAO in SHR than in WKY, which may result from an increased GBB permeability to TMA and thereby increased TMA delivery to the liver in SHR.

In conclusion, the present study shows that hypertension in rats is associated with an increased permeability of the colon to TMA, a gut bacterial metabolite, which is accompanied by morphological and hemodynamic alterations in the colon. This suggests that hypertension increases the penetration of gut microbiota products to the circulation.

Several clinical trials showed a positive correlation between an elevated plasma TMAO level and an increased cardiovascular risk. Furthermore, it has been speculated that an increased plasma concentration of TMAO may contribute to the etiology and symptoms of cardiovascular diseases. Our findings imply that high plasma TMAO concentration in cardiovascular diseases may depend on increased permeability of the colon to TMA, a TMAO precursor. Therefore, the alterations in the colon permeability, but not plasma TMAO level, may be a marker of cardiovascular risk.

## References

[1] Ufnal M, Jazwiec R, Dadlez M, Drapala A, Sikora M, Skrzypecki J. Trimethylamine-N-oxide: a carnitine-derived metabolite that prolongs the hypertensive effect of angiotensin II in rats. The Canadian journal of cardiology. 2014;30(12):1700–5. doi: 10.1016/j.cjca.2014.09.010 . 25475471 10.1016/j.cjca.2014.09.010

[2] Tomasova L, Konopelski P, Ufnal M. Gut Bacteria and Hydrogen Sulfide: The New Old Players in Circulatory System Homeostasis. Molecules. 2016;21(11). doi: 10.3390/molecules21111558 . 27869680 10.3390/molecules21111558 PMC6273628

[3] Wang Z, Tang WH, Buffa JA, Fu X, Britt EB, Koeth RA, et al Prognostic value of choline and betaine depends on intestinal microbiota-generated metabolite trimethylamine-N-oxide. European heart journal. 2014;35(14):904–10. doi: 10.1093/eurheartj/ehu002 ; PubMed Central PMCID: PMC3977137. 24497336 10.1093/eurheartj/ehu002 PMC3977137

[4] Matheson PJ, Wilson MA, Garrison RN. Regulation of intestinal blood flow. The Journal of surgical research. 2000;93(1):182–96. doi: 10.1006/jsre.2000.5862 . 10945962 10.1006/jsre.2000.5862

[5] Schmieder RE. End organ damage in hypertension. Deutsches Arzteblatt international. 2010;107(49):866–73. doi: 10.3238/arztebl.2010.0866 ; PubMed Central PMCID: PMC3011179. 21191547 10.3238/arztebl.2010.0866 PMC3011179

[6] Senthong V, Wang Z, Li XS, Fan Y, Wu Y, Tang WH, et al Intestinal Microbiota-Generated Metabolite Trimethylamine-N-Oxide and 5-Year Mortality Risk in Stable Coronary Artery Disease: The Contributory Role of Intestinal Microbiota in a COURAGE-Like Patient Cohort. Journal of the American Heart Association. 2016;5(6). doi: 10.1161/JAHA.115.002816 ; PubMed Central PMCID: PMC4937244. 27287696 10.1161/JAHA.115.002816 PMC4937244

[7] Nowinski A. Ufnal M. Trimethylamine N-oxide: A harmful, protective or diagnostic marker in lifestyle diseases? Nutrition. 2018;46:7–12. doi: 10.1016/j.nut.2017.08.001 10.1016/j.nut.2017.08.001 29290360

[8] Kühn T, Rohrmann S, Sookthai D, Johnson T, Katzke V, Kaaks R, et al Intra-individual variation of plasma trimethylamine-N-oxide (TMAO), betaine and choline over 1 year. Clinical Chemistry and Laboratory Medicine (CCLM). 2017;55(2):261–8. 27447240 10.1515/cclm-2016-0374

[9] Zeisel SH, Wishnok JS, Blusztajn JK. Formation of methylamines from ingested choline and lecithin. The Journal of pharmacology and experimental therapeutics. 1983;225(2):320–4. . 6842395

[10] Koeth RA, Wang Z, Levison BS, Buffa JA, Org E, Sheehy BT, et al Intestinal microbiota metabolism of L-carnitine, a nutrient in red meat, promotes atherosclerosis. Nature medicine. 2013;19(5):576–85. doi: 10.1038/nm.3145 ; PubMed Central PMCID: PMC3650111. 23563705 10.1038/nm.3145 PMC3650111

[11] Wang Z, Klipfell E, Bennett BJ, Koeth R, Levison BS, Dugar B, et al Gut flora metabolism of phosphatidylcholine promotes cardiovascular disease. Nature. 2011;472(7341):57–63. doi: 10.1038/nature09922 ; PubMed Central PMCID: PMC3086762. 21475195 10.1038/nature09922 PMC3086762

[12] Bain MA, Fornasini G, Evans AM. Trimethylamine: metabolic, pharmacokinetic and safety aspects. Current drug metabolism. 2005;6(3):227–40. . 15975041 10.2174/1389200054021807

[13] Mansoor JK, Schelegle ES, Davis CE, Walby WF, Zhao W, Aksenov AA, et al Analysis of volatile compounds in exhaled breath condensate in patients with severe pulmonary arterial hypertension. PloS one. 2014;9(4):e95331 doi: 10.1371/journal.pone.0095331 ; PubMed Central PMCID: PMC3991617. 24748102 10.1371/journal.pone.0095331 PMC3991617

[14] Schindelin J, Arganda-Carreras I, Frise E, Kaynig V, Longair M, Pietzsch T, et al Fiji: an open-source platform for biological-image analysis. Nature methods. 2012;9(7):676–82. doi: 10.1038/nmeth.2019 ; PubMed Central PMCID: PMC3855844. 22743772 10.1038/nmeth.2019 PMC3855844

[15] Tomasova L, Dobrowolski L, Jurkowska H, Wrobel M, Huc T, Ondrias K, et al Intracolonic hydrogen sulfide lowers blood pressure in rats. Nitric oxide: biology and chemistry. 2016;60:50–8. doi: 10.1016/j.niox.2016.09.007 . 27667183 10.1016/j.niox.2016.09.007

[16] Li XS, Obeid S, Klingenberg R, Gencer B, Mach F, Raber L, et al Gut microbiota-dependent trimethylamine N-oxide in acute coronary syndromes: a prognostic marker for incident cardiovascular events beyond traditional risk factors. European heart journal. 2017 doi: 10.1093/eurheartj/ehw582 . 28077467 10.1093/eurheartj/ehw582 PMC5837488

[17] Zhu W, Gregory JC, Org E, Buffa JA, Gupta N, Wang Z, et al Gut Microbial Metabolite TMAO Enhances Platelet Hyperreactivity and Thrombosis Risk. Cell. 2016;165(1):111–24. doi: 10.1016/j.cell.2016.02.011 ; PubMed Central PMCID: PMC4862743. 26972052 10.1016/j.cell.2016.02.011 PMC4862743

[18] Yancey PH, Siebenaller JF. Co-evolution of proteins and solutions: protein adaptation versus cytoprotective micromolecules and their roles in marine organisms. The Journal of experimental biology. 2015;218(Pt 12):1880–96. doi: 10.1242/jeb.114355 . 26085665 10.1242/jeb.114355

[19] Ufnal M, Pham K. The gut-blood barrier permeability—A new marker in cardiovascular and metabolic diseases? Medical hypotheses. 2017;98:35–7. doi: 10.1016/j.mehy.2016.11.012 . 28012600 10.1016/j.mehy.2016.11.012

[20] Farhadi A, Banan A, Fields J, Keshavarzian A. Intestinal barrier: an interface between health and disease. Journal of gastroenterology and hepatology. 2003;18(5):479–97. . 12702039 10.1046/j.1440-1746.2003.03032.x

[21] Groschwitz KR, Hogan SP. Intestinal barrier function: molecular regulation and disease pathogenesis. The Journal of allergy and clinical immunology. 2009;124(1):3–20; quiz 1–2. doi: 10.1016/j.jaci.2009.05.038 ; PubMed Central PMCID: PMC4266989. 19560575 10.1016/j.jaci.2009.05.038 PMC4266989

[22] Sandek A, Bauditz J, Swidsinski A, Buhner S, Weber-Eibel J, von Haehling S, et al Altered intestinal function in patients with chronic heart failure. Journal of the American College of Cardiology. 2007;50(16):1561–9. doi: 10.1016/j.jacc.2007.07.016 . 17936155 10.1016/j.jacc.2007.07.016

[23] Santisteban MM, Qi Y, Zubcevic J, Kim S, Yang T, Shenoy V, et al Hypertension-Linked Pathophysiological Alterations in the Gut. Circulation research. 2017;120(2):312–23. doi: 10.1161/CIRCRESAHA.116.309006 ; PubMed Central PMCID: PMC5250568. 27799253 10.1161/CIRCRESAHA.116.309006 PMC5250568

[24] Meijers BK, Evenepoel P. The gut-kidney axis: indoxyl sulfate, p-cresyl sulfate and CKD progression. Nephrology, dialysis, transplantation: official publication of the European Dialysis and Transplant Association—European Renal Association. 2011;26(3):759–61. doi: 10.1093/ndt/gfq818 . 21343587 10.1093/ndt/gfq818

[25] Washington C, Carmichael JC. Management of ischemic colitis. Clinics in colon and rectal surgery. 2012;25(4):228–35. doi: 10.1055/s-0032-1329534 ; PubMed Central PMCID: PMC3577613. 24294125 10.1055/s-0032-1329534 PMC3577613

[26] Fink MP, Kaups KL, Wang HL, Rothschild HR. Maintenance of superior mesenteric arterial perfusion prevents increased intestinal mucosal permeability in endotoxic pigs. Surgery. 1991;110(2):154–60; discussion 60–1. . 1907030

[27] Thuijls G, de Haan JJ, Derikx JP, Daissormont I, Hadfoune M, Heineman E, et al Intestinal cytoskeleton degradation precedes tight junction loss following hemorrhagic shock. Shock. 2009;31(2):164–9. doi: 10.1097/SHK.0b013e31817fc310 . 18650780 10.1097/SHK.0b013e31817fc310

[28] Mishra RC, Tripathy S, Gandhi JD, Balsevich J, Akhtar J, Desai KM, et al Decreases in splanchnic vascular resistance contribute to hypotensive effects of L-serine in hypertensive rats. American journal of physiology Heart and circulatory physiology. 2010;298(6):H1789–96. doi: 10.1152/ajpheart.00810.2009 . 20348218 10.1152/ajpheart.00810.2009

[29] Sawada N. Tight junction-related human diseases. Pathology international. 2013;63(1):1–12. doi: 10.1111/pin.12021 . 23356220 10.1111/pin.12021 PMC7168075

[30] Miele L, Valenza V, La Torre G, Montalto M, Cammarota G, Ricci R, et al Increased intestinal permeability and tight junction alterations in nonalcoholic fatty liver disease. Hepatology. 2009;49(6):1877–87. doi: 10.1002/hep.22848 . 19291785 10.1002/hep.22848

